# Supplementary material for: Effects of Dietary Nano-Zinc Oxide Supplementation on Meat Quality, Antioxidant Capacity and Cecal Microbiota of Intrauterine Growth Retardation Finishing Pigs
Source: Foods. 2023 May 4;12(9):1885. doi: 10.3390/foods12091885 (PMC10178521; doi:10.3390/foods12091885)
Supplement: Supplementary file 1 [file foods-12-01885-s001.zip › foods-2312769-supplementary.pdf]

Table S1. Composition and nutrient levels of basal diets for experimental pigs.

Items	Content		
	21-56 d	57-77 d	78-163 d
Ingredient (%)			
Corn	24.00	65.00	62.50
Wheat middling	5.00	8.00	13.00
Extruded Soybean	5.00	-	-
Soybean meal	-	21.00	18.00
Fermented soybean meal	8.00	-	-
Broken rice	16.81	-	-
Fish meal	3.00	-	-
Milk powder	10.00	-	-
Whey powder	10.00	-	-
Soyben protein concentrate	4.00	-	-
Yeast	1.00	-	-
Soybean oil	0.65	2.00	2.50
Cane sugar	3.00	-	-
Glucose	4.00	-	-
Dicalcium phosphate	0.74	-	-
Limestone	0.80	-	-
Premix[1]	4.00	4.00	4.00
Total	100.00	100.00	100.00
Calculated Nutrient levels[2]			
Digestible energy (MJ/Kg)	14.90	14.07	14.16
Crude protein (%)	19.02	16.97	16.03
Lysine (%)	1.32	0.99	0.93
Methionine + cysteine (%)	0.78	0.68	0.66
Threonine (%)	0.83	0.69	0.65
P (%)	0.43	0.28	0.27
Ca (%)	0.87	0.64	0.63
Zn (mg/kg)	180.89	139.46	133.41

[1]Premix provided per kilogram of basal diet:

For 21-56 d: Lys (78.8%), 2.50 g; Met (99%), 1.50g; Thr (98%), 0.50 g; NaCL,3.00 g; vitamin E, 80 IU; vitamin A, 10000 IU; vitamin D_3, 1500 IU; vitamin K_3, 3.0 mg; biotin, 0.20 mg; niacin, 25 mg; D-pantothenic acid, 30 mg; riboflavin, 3.6 mg; thiamine, 1.0 mg; pyridoxin, 1.5 mg; folic acid, 2.0 mg; choline, 800 mg; Fe (FeSO4), 120 mg; Zn (ZnSO4), 100 mg; Mn, 80 mg; Cu, 25 mg; I, 0.30 mg; Se, 0.25 mg. Multi-enzyme complex (β-glucanase, 100 U; xylanase, 200 U and phytase, 200 U).

For 57-77 d: Lys(78.8%), 2.50 g; Met (99%), 1.50g; Thr (98%), 0.50 g; NaCL3.00 g, Limestone, 10g; Dicalcium phosphate, 9.0g; vitamin E, 40 IU; vitamin A, 3000 IU; vitamin D_3, 1000 IU; vitamin K_3, 1.50 mg; biotin, 0.20 mg; niacin, 20 mg; D-pantothenic acid, 25 mg; riboflavin, 3.5 mg; thiamine, 1.0 mg; pyridoxin, 1.50 mg; folic acid, 1.0 mg; choline, 500 mg; Fe, 110 mg; Zn (ZnSO4), 100 mg; Mn, 40 mg; Cu, 15 mg; I, 0.25 mg; Se, 0.20 mg. Multi-enzyme complex (β-glucanase, 300 U; xylanase, 500 U and phytase , 500 U).

For 78-163 d: Lys(78.8%), 2.50 g; Met (99%), 1.50g; Thr (98%), 0.50 g; NaCL3.00 g, Limestone, 10.00g; Dicalcium phosphate, 8.50g; vitamin E, 40 IU; vitamin A, 3000 IU; vitamin D_3, 1000 IU; vitamin K_3, 1.50 mg; biotin, 0.20 mg; niacin, 20 mg; D-pantothenic acid, 25 mg; riboflavin, 3.5 mg; thiamine, 1.0 mg; pyridoxin, 1.50 mg; folic acid, 1.0 mg; choline, 500 mg; Fe, 110 mg; Zn (ZnSO4), 100 mg; Mn, 40 mg; Cu, 15 mg; I, 0.25 mg; Se, 0.20 mg.

[2] Nutrient levels were calculated values, except for Zn concentration, which was analyzed via fame

atomic absorption spectrometry.

Table S2. Sequences for real-time PCR primers.

Gene name[1]	Accession No	Primer sequence (5′ to 3′)	Product size (bp)
MyHc Ⅰ a	NM_213855.2	F: ACCAACCTGTCCAAGTTCCG<br>R: AGGACTGGGAGCTTTGTTGC	193
MyHc Ⅱ a	NM_214136.1	F: GGACCCCCTGAATGACACAG<br>R: CGGTCTGGAAGGAAGAACCC	149
MyHc Ⅱ x	NM_001104951.2	F: ACATTACTGGCTGGCTGGAC<br>R: CTTTCCACCTCCAGCCTCTG	140
MyHc Ⅱ b	NM_001123141.1	F: AGGAGCATCAGCGCCTAATC<br>R: TCGGGATAGCTGAGACACCA	119
Nrf2	XM_013984303.2	F: ATCCAGCGGATTGCTCGTAG<br>R: TCAAATCCATGTCCTTGGCG	155
Keap1	NM_001114671.1	F: TCTGCTTAGTCATGGTGACCT<br>R: GGGGTTCCAGATGACAAGGG	158
GCLM	XM_001926378.4	F: GGACAAAACCCAGTTGGAGC<br>R: TCACACAGCAAGAGGCAAGA	86
GCLC	XM_003482164.4	F: CTAGTGGGTAGGCGGACTGG<br>R: CGGTGTCGTGCTCTAGCTTC	81
HO-1	NM_001004027.1	F: CAAGCAGAAAATCCTCGAAG<br>R: GCTGAGTGTCAGGACCCATC	241
GPX1	NM_214201.1	F: CCTCAAGTACGTCCGACCAG<br>R: GTGAGCATTTGCGCCATTCA	85
GPX4	NM_214407.1	F: CACCCTCTGTGGAAGTGGAT<br>R: TCACCACACAGCCGTTCTTA	112
NQO1	NM_001159613.1	F: GATCATACTGGCCCACTCCG	200
GR	AY368271.1	F: GTGAGCCGACTGAACACCAT<br>R: CAGGATGTGAGGAGCTGTGT	141
GSTA1	NM_214389.2	F: CAGGACACCCAGGACCAATC<br>R: GTCTCAGGTACATTCCGGGAG	202
GSTT1	NM_001315568.1	F: GCAGAAGGTGCCTGTCTTGA<br>R: AGGCCAGGTACTCATCCACT	149
GSTK1	NM_001315574.1	F: TACGATGTGCTGTCCCCCTA<br>R: ATAGCTGGTGGCTGGTTTCC	140
GAPDH	NM_001206359.1	F: ATGGTGAAGGTCGGAGTGAA<br>R: CCGTGGGTGGAATCATACTG	155

MyHc, myosin heavy chain; Nrf2, Nuclear factor erythroid-derived 2-like 2; Keap1, Kelch-like ECH-associated protein 1; GCLM, glutamate-cysteine ligase modifer subunit; GCLC, glutamate-cysteine ligase catalytic subunit; HO-1, heme oxygenase 1; GPX, glutathione peroxidase; NQO1, NAD(P)H dehydrogenase, quinone 1; GR, glutathione reductase; GSTA1, glutathione S-transferase alpha 1; GSTT1, glutathione S-transferase theta-1; GSTK1, glutathione S-transferase kappa 1.
